# Supplementary material for: Analysis of Raffinose Synthase Gene Family in Bread Wheat and Identification of Drought Resistance and Salt Tolerance Function of TaRS15-3B
Source: Int J Mol Sci. 2023 Jul 6;24(13):11185. doi: 10.3390/ijms241311185 (PMC10342549; doi:10.3390/ijms241311185)
Supplement: Supplementary file 1 [file ijms-24-11185-s001.zip › ijms-2460919-supplementary.pdf]

Supplementary Materials

Table S1 The basic information of 34 wheat TaRS genes.

Gene name	Transcript stable ID	Subfamily	Amino size	MW	pI	Instability Index	Aliphatic Index	Grand Average of Hydropathicity	Predicting subcellular localization
TaRS2-1A	TraesCS1A02G103500.1	Ⅳ	402	43995.67	8.55	34.71	75.25	-0.366	chlo, extr, vacu
TaRS4-1A	TraesCS1A02G103600.1	Ⅳ	846	91460.44	5.2	35.9	79.59	-0.085	cyto, nucl, chlo, plas, extr
TaRS1-1A	TraesCS1A02G164800.1	Ⅳ	314	34727.87	7.61	35	66.56	-0.518	mito, chlo, nucl., nucl_plas
TaRS3-1A	TraesCS1A02G434900.1	Ⅲ	373	41339.35	6.07	37.31	67.53	-0.431	chlo, nucl, mito, cyto, plas
TaRS5-1B	TraesCS1B02G181800.1	Ⅳ	371	41139	6.07	37.61	67.39	-0.439	chlo, nucl., cyto, nucl_plas.
TaRS7-1D	TraesCS1D02G161900.1	Ⅳ	98	10759.32	9.73	48.02	72.86	-0.19	pero, nucl, chlo, cyto
TaRS6-1D	TraesCS1D02G294900.1	Ⅰ	411	44693.02	7.04	34.57	71.31	-0.414	vacu, chlo, extr, golg, nucl
TaRS8-1D	TraesCS1D02G444000.1	Ⅲ	799	86503.86	5.2	35.12	80.48	-0.084	cyto, nucl, chlo, extr, plas
TaRS10-2A	TraesCS2A02G284600.1	Ⅰ	767	83539.61	5.25	38.41	86.35	-0.103	chlo, cyto, nucl, plas, E.R._vacu
TaRS9-2A	TraesCS2A02G324700.1	Ⅱ	98	10947.55	9.5	51.31	71.84	-0.222	pero, nucl, chlo, cyto
TaRS11-2B	TraesCS2B02G357600.1	Ⅱ	1119	120845.26	8.43	49.07	80.88	-0.227	cyto, nucl, mito
TaRS12-2D	TraesCS2D02G338200.1	Ⅱ	767	83608.63	5.24	37.65	85.7	-0.126	chlo, cyto, nucl, plas
TaRS13-3A	TraesCS3A02G092800.1	Ⅲ	782	85073.87	5.51	33.33	85.28	-0.067	cyto, nucl, plas, chlo, E.R._vacu
TaRS14-3A	TraesCS3A02G113900.1	Ⅰ	752	81994.98	5.76	34.07	80.51	-0.173	cyto, chlo, mito
TaRS16-3B	TraesCS3B02G108000.1	Ⅲ	756	82523.35	5.71	33.4	78.92	-0.217	cyto, mito

Table S1（continued）

Gene name	Transcript stable ID	Subfamily	Amino size	MW	pI	Instability Index	Aliphatic Index	Grand Average of Hydropathicity	Predicting subcellular localization
TaRS15-3B	TraesCS3B02G133400.1	Ⅰ	782	85005.78	5.36	33.92	84.51	-0.074	cyto, nucl, plas, chlo, E.R._vacu
TaRS18-3D	TraesCS3D02G092900.1	Ⅲ	787	85849.38	5.64	35.36	81.37	-0.152	chlo, vacu, E.R., cyto., cyto_nucl., mito
TaRS17-3D	TraesCS3D02G116400.1	Ⅰ	885	96173.5	5.78	34.41	84.38	-0.078	cyto, chlo, nucl, plas, E.R._vacu
TaRS19-4A	TraesCS4A02G174200.1	Ⅰ	98	10860.38	9.25	61.23	70.92	-0.266	pero, nucl, cyto
TaRS20-5A	TraesCS5A02G173200.1	Ⅳ	387	42593.86	5.14	30.43	76.98	-0.349	chlo, extr, mito, E.R.
TaRS23-5B	TraesCS5B02G011700.1	Ⅳ	387	42593.78	5.33	30.92	74.42	-0.383	chlo, extr, mito
TaRS21-5B	TraesCS5B02G011800.1	Ⅳ	402	44009.78	8.83	34.04	73.58	-0.36	chlo, mito, E.R.
TaRS22-5B	TraesCS5B02G170600.1	Ⅳ	401	44028.66	8.56	33.67	75.49	-0.353	vacu, chlo, extr, nucl, mito, golg
TaRS25-5D	TraesCS5D02G019100.1	Ⅳ	387	42686.93	5.25	28.77	75.17	-0.397	chlo, extr, mito
TaRS26-5D	TraesCS5D02G177700.1	Ⅳ	98	10829.41	9.73	51.7	70.82	-0.23	pero, nucl, chlo, cyto
TaRS24-5D	TraesCS5D02G271300.1	Ⅰ	401	44161.88	8.37	31.53	78.15	-0.326	vacu, chlo, golg, nucl, mito, extr, E.R.
TaRS27-5D	TraesCS5D02G300200.1	Ⅰ	98	10877.52	9.66	57.83	69.9	-0.207	pero, nucl, chlo, cyto
TaRS28-7A	TraesCS7A02G134100.1	Ⅰ	856	90934.31	6.06	44.49	79.6	-0.103	chlo, mito
TaRS30-7A	TraesCS7A02G236500.1	Ⅰ	98	10802.45	10.03	64.74	66.94	-0.256	pero, nucl, chlo, cyto
TaRS29-7A	TraesCS7A02G503400.1	Ⅰ	749	81213.59	5.77	31.78	78.76	-0.184	cyto, nucl, plas, cysk, E.R._vacu
TaRS32-7B	TraesCS7B02G035200.1	Ⅰ	749	81421.8	5.65	32.34	79.13	-0.197	cyto, cysk, plas
TaRS31-7B	TraesCS7B02G134600.1	Ⅰ	804	85250.95	6.02	40.07	84.45	-0.01	cyto, chlo, nucl, mito
TaRS33-7D	TraesCS7D02G133500.1	Ⅰ	798	85079.8	6.25	38.24	84.12	-0.018	cyto, chlo, plas, nucl, mito
TaRS34-7D	TraesCS7D02G236700.1	Ⅰ	749	81087.62	5.75	33.17	79.4	-0.162	cyto, nucl, plas, cysk,

Table S2 The number of *RS* genes found in each subfamily of *Triticum aestivum*、*Aegilops tauschii Coss*、*Oryza sativa Japonica*、*Zea mays*、*Arabidopsis thaliana*、*Glycine.max*、*Gossypium raimondii* 、*Solanum tuberosum* L. and *Solanum lycopersicum* L.

Plant class	species	Number of genes			
		RS I	RSII	RS III	RS IV
Monocotyledons	Triticum aestivum	15	3	5	11
	Aegilops tauschii Coss	6	1	2	10
	Oryza sativa Japonica	6	1	1	4
	Zea mays	4	5	1	9
Dicotyledons	Arabidopsis thaliana	3	0	2	3
	Gossypium raimondii	13	8	5	13
	Glycine.max	9	3	6	18
	Solanum tuberosum L.	6	7	3	6
	Solanum lycopersicum L.	3		3	3

Table S3 The precise location of TaRS gene on wheat chromosomes and homologous gene pairs

Gene name	Position	Homologs
TaRS2-1A	1A:99657553-99659873	TaRS4-1A、TaRS21-5B、TaRS25-5D
TaRS4-1A	1A:99697964-99701912	TaRS2-1A、TaRS23-5B、TaRS7-1D
TaRS1-1A	1A:297744935-297749437	TaRS5-1B、TaRS7-1D、TaRS2-1A
TaRS3-1A	1A:585615984-585620070	TaRS8-1D
TaRS5-1B	1B:328820458-328824835	TaRS1-1A、TaRS7-1D、TaRS23-5B
TaRS7-1D	1D:229483469-229489471	TaRS1-1A、TaRS5-1B、TaRS25-5D
TaRS6-1D	1D:393950537-393951163	TaRS29-7A、TaRS24-5D
TaRS8-1D	1D:487544924-487548208	TaRS3-1A
TaRS10-2A	2A:478456590-478457216	TaRS27-5D
TaRS9-2A	2A:554737170-554741912	TaRS11-2B、TaRS12-2D
TaRS11-2B	2B:510588121-510591237	TaRS9-2A、TaRS12-2D
TaRS12-2D	2D:431676823-431681015	TaRS9-2A、TaRS11-2B
TaRS13-3A	3A:58836094-58839205	TaRS16-3B、TaRS18-3D
TaRS14-3A	3A:81475905-81479675	TaRS15-3B、TaRS17-3D
TaRS16-3B	3B:73627626-73630835	TaRS13-3A、TaRS18-3D
TaRS15-3B	3B:115780571-115784482	TaRS14-3A、TaRS17-3D
TaRS18-3D	3D:47088455-47090932	TaRS13-3A、TaRS16-3B
TaRS17-3D	3D:69854131-69857808	TaRS14-3A、TaRS15-3B
TaRS19-4A	4A:444378778-444379403	NA
TaRS20-5A	5A:366629867-366633348	TaRS22-5B、TaRS26-5D
TaRS23-5B	5B:11513383-11517316	TaRS4-1A、TaRS21-5B、TaRS25-5D
TaRS21-5B	5B:11550484-11556238	TaRS2-1A、TaRS23-5B、TaRS25-5D
TaRS22-5B	5B:315609978-315613753	TaRS20-5A、TaRS26-5D
TaRS25-5D	5D:11431758-11435533	TaRS2-1A、TaRS21-5B、TaRS7-1D
TaRS26-5D	5D:277555927-277559625	TaRS20-5A、TaRS22-5B
TaRS24-5D	5D:375002992-375003618	TaRS6-1D、TaRS29-7A
TaRS27-5D	5D:397471556-397472182	TaRS29-7A、TaRS24-5D
TaRS28-7A	7A:85911103-85915219	TaRS32-7B、TaRS33-7D
TaRS30-7A	7A:209693821-209698303	TaRS31-7B、TaRS34-7D
TaRS29-7A	7A:692230361-692230987	TaRS6-1D
TaRS32-7B	7B:34387130-34389855	TaRS28-7A、TaRS33-7D
TaRS31-7B	7B:164585536-164590079	TaRS30-7A、TaRS34-7D
TaRS33-7D	7D:84600233-84604354	TaRS28-7A、TaRS32-7B
TaRS34-7D	7D:199626866-199631358	TaRS30-7A、TaRS31-7B

Table S4 The non redundant collinearity genes of different species and their Ka, Ks and Ka/Ks in the TaRS gene family of wheat

Gene 1	Gene2	Ka	Ks	Ka/Ks
TaRS4-1A	TaRS8-1D	0.034802	0.129431	0.268886
TaRS3-1A	TaRS6-1D	0.005325	0.083622	0.063678
TaRS5-1B	TaRS6-1D	0.005323	0.068454	0.077766
TaRS10-2A	TaRS11-2B	0.013717	0.073593	0.186385
TaRS10-2A	TaRS6-1D	0.012166	0.091464	0.133014
TaRS13-3A	TaRS6-1D	0.017768	0.087598	0.202842
TaRS14-3A	TaRS16-3B	0.019018	0.152141	0.125
TaRS13-3A	TaRS17-3D	0.018656	0.107887	0.17292
TaRS14-3A	TaRS18-3D	0.022821	0.12715	0.17948
TaRS15-3B	TaRS17-3D	0.008837	0.088404	0.099957
TaRS16-3B	TaRS18-3D	0.023276	0.102874	0.226259
TaRS20-5A	TaRS23-5B	0.022098	0.060778	0.363585
TaRS20-5A	TaRS25-5D	0.015824	0.054375	0.29102
TaRS23-5B	TaRS25-5D	0.01523	0.028785	0.529106
TaRS21-5B	TaRS24-5D	0.042949	0.208296	0.20619
TaRS28-7A	TaRS31-7B	0.026002	0.06883	0.377778
TaRS29-7A	TaRS32-7B	0.019933	0.088696	0.22473
TaRS28-7A	TaRS33-7D	0.025803	0.073456	0.351267
TaRS29-7A	TaRS34-7D	0.013341	0.105125	0.126905
TaRS32-7B	TaRS34-7D	0.016631	0.100017	0.166282
TaRS32-7B	AT1G55740.1	0.21054	NaN	NaN
TaRS34-7D	AT1G55740.1	0.213935	NaN	NaN
TaRS4-1A	AET1Gv21025900.1	0.040373	0.136843	0.295035
TaRS6-1D	AET1Gv20424800.7	0	0.00345	0
TaRS8-1D	AET1Gv21025900.1	6.87E-04	0	#NAME?
TaRS10-2A	AET2Gv20764600.1	0.012166	0.093537	0.130066
TaRS13-3A	AET3Gv20201400.1	0.016912	0.09808	0.172429
TaRS15-3B	AET3Gv20201400.1	0.007117	0.084584	0.084143
TaRS16-3B	AET3Gv20252200.1	0.023276	0.102874	0.226259
TaRS18-3D	AET3Gv20252200.1	0	0	NaN
TaRS20-5A	AET5Gv20434400.4	0.129917	0.137609	0.944102
TaRS21-5B	AET5Gv20036400.3	0.043482	0.206982	0.210077
TaRS23-5B	AET5Gv20434400.4	0.12259	0.099738	1.229117
TaRS24-5D	AET5Gv20036400.3	0	0	NaN
TaRS25-5D	AET5Gv20434400.4	0.123178	0.097661	1.261286
TaRS27-5D	AET5Gv20682600.1	0	0	NaN
TaRS28-7A	AET7Gv20338600.2	0.030422	0.088871	0.342315
TaRS29-7A	AET7Gv20586000.6	0.013341	0.105125	0.126905
TaRS32-7B	AET7Gv20586000.6	0.016631	0.100017	0.166282
TaRS34-7D	AET7Gv20586000.6	0	0	NaN
TaRS10-2A	KRH63661	0.312082	4.253764	0.073366

Table S4 （continued）

Gene 1	Gene2	Ka	Ks	Ka/Ks
TaRS10-2A	KRH54286	0.327436	NaN	NaN
TaRS13-3A	KRH57114	0.316905	3.432716	0.092319
TaRS13-3A	KRH54343	0.283557	NaN	NaN
TaRS15-3B	KRH57114	0.310907	NaN	NaN
TaRS15-3B	KRH54343	0.278225	NaN	NaN
TaRS28-7A	KRH20155	0.256039	1.545269	0.165692
TaRS28-7A	KRH03657	0.261119	1.659674	0.157332
TaRS34-7D	KRH36658	0.47402	NaN	NaN
TaRS6-1D	KJB79780	0.277668	NaN	NaN
TaRS10-2A	KJB19525	0.365706	NaN	NaN
TaRS29-7A	KJB81540	0.184247	NaN	NaN
TaRS32-7B	KJB63105	0.220068	NaN	NaN
TaRS34-7D	KJB81540	0.184617	NaN	NaN
TaRS34-7D	KJB63105	0.220912	NaN	NaN
TaRS6-1D	Os10t0492900-01	0.294388	1.330272	0.221299
TaRS10-2A	Os04t0481100-02	0.131099	0.491769	0.266587
TaRS13-3A	Os01t0170000-01	0.120371	0.297019	0.405264
TaRS15-3B	Os01t0170000-01	0.117591	0.267137	0.44019
TaRS28-7A	Os06t0172800-00	0.166157	0.595842	0.278861
TaRS29-7A	Os08t0495800-02	0.082413	0.689327	0.119556
TaRS32-7B	Os08t0495800-02	0.078448	0.646026	0.121431
TaRS34-7D	Os08t0495800-02	0.079065	0.679402	0.116375
TaRS13-3A	Solyc03g112500.3.1	0.120371	NaN	NaN
TaRS15-3B	Solyc03g112500.3.1	0.082413	NaN	NaN
TaRS32-7B	Solyc07g065980.3.1	0.184617	NaN	NaN
TaRS10-2A	PGSC0003DMT400039195	0.317026	NaN	NaN
TaRS13-3A	PGSC0003DMT400046636	0.266292	NaN	NaN
TaRS32-7B	PGSC0003DMT400057320	0.214123	NaN	NaN
TaRS34-7D	PGSC0003DMT400057320	0.212059	NaN	NaN
TaRS6-1D	Zm00001eb044840_T001	0.103935	0.813602	0.127746
TaRS10-2A	Zm00001eb079430_T001	0.134927	0.543573	0.248223
TaRS13-3A	Zm00001eb122980_T001	0.110372	0.384147	0.287317
TaRS15-3B	Zm00001eb122980_T001	0.103905	0.392396	0.264796
TaRS28-7A	Zm00001eb281720_T002	0.147354	0.382349	0.385393
TaRS29-7A	Zm00001eb033870_T003	0.106151	0.857501	0.123791
TaRS32-7B	Zm00001eb033870_T003	0.10593	0.909002	0.116535
TaRS34-7D	Zm00001eb033870_T003	0.106616	0.892232	0.119493

Table S5 GO Enrichment Analysis of TaRS

Gene name	GO Term
TraesCS1A02G164800	GO:0005737 GO:0007020 GO:0052692 GO:0009620 GO:0009911 GO:0009965 GO:0016139 GO:0048046 GO:0071555 GO:0046477 GO:0000023 GO:0009505 GO:0043085 GO:0019252
TraesCS5B02G011800	GO:0005737 GO:0007020 GO:0052692 GO:0009620 GO:0009911 GO:0009965 GO:0016139 GO:0048046 GO:0071555 GO:0046477 GO:0000023 GO:0009505 GO:0043085 GO:0019252
TraesCS3B02G133400	GO:0047274 GO:0005737 GO:0052692 GO:0034484 GO:0080167 GO:0009409 GO:0006979 GO:0009506
TraesCS5B02G011700	GO:0005737 GO:0007020 GO:0052692 GO:0009620 GO:0009911 GO:0009965 GO:0016139 GO:0048046 GO:0071555 GO:0046477 GO:0000023 GO:0009505 GO:0043085 GO:0019252
TraesCS1A02G434900	GO:0005737 GO:0047274 GO:0005975 GO:0047268 GO:0080167 GO:0004553 GO:0006979
TraesCS7D02G236700	GO:0047274 GO:0018107 GO:0052692 GO:0018105 GO:0009737 GO:0009414 GO:0004674 GO:0005986 GO:0010325 GO:0034484 GO:0080167 GO:0005886 GO:0009409 GO:0035556 GO:0009507 GO:0006979 GO:0005524 GO:0019593
TraesCS2A02G284600	GO:0003824 GO:0008152
TraesCS7A02G236500	GO:0047274 GO:0018107 GO:0052692 GO:0018105 GO:0009737 GO:0009414 GO:0004674 GO:0005986 GO:0010325 GO:0034484 GO:0080167 GO:0005886 GO:0009409 GO:0035556 GO:0009507 GO:0006979 GO:0005524 GO:0019593
TraesCS1A02G103600	GO:0005737 GO:0007020 GO:0052692 GO:0009620 GO:0009911 GO:0009965 GO:0016139 GO:0048046 GO:0071555 GO:0046477 GO:0000023 GO:0009505 GO:0043085 GO:0019252
TraesCS2A02G324700	GO:0047274 GO:0052692 GO:0034484 GO:0080167 GO:0009409 GO:0006979
TraesCS2B02G357600	GO:0047274 GO:0052692 GO:0034484 GO:0080167 GO:0009409 GO:0006979
TraesCS1B02G181800	GO:0052692 GO:0009965 GO:0016139 GO:0048046 GO:0032940 GO:0050825 GO:0050826 GO:0000023 GO:0043085 GO:0007020 GO:0031410 GO:0016020 GO:0071555 GO:0017153 GO:0019252 GO:0006835 GO:0042309 GO:0009505 GO:0009620 GO:0009911 GO:0005515 GO:0046477
TraesCS1D02G161900	GO:0005737 GO:0007020 GO:0052692 GO:0009620 GO:0009911 GO:0009965 GO:0016139 GO:0048046 GO:0071555 GO:0046477 GO:0000023 GO:0009505 GO:0043085 GO:0019252
TraesCS5D02G019100	GO:0005737 GO:0007020 GO:0052692 GO:0009620 GO:0009911 GO:0009965 GO:0016139 GO:0048046 GO:0071555 GO:0046477 GO:0000023 GO:0009505 GO:0043085 GO:0019252
TraesCS1A02G103500	GO:0005737 GO:0007020 GO:0052692 GO:0005515 GO:0009620 GO:0009911 GO:0009965 GO:0016139 GO:0048046 GO:0071555 GO:0050825 GO:0042309 GO:0050826 GO:0046477 GO:0000023 GO:0009505 GO:0043085 GO:0019252

Table S5（continued）

Gene name	GO Term
TraesCS7A02G134100	GO:0003824 GO:0008152
TraesCS3D02G092900	GO:0047274 GO:0018107 GO:0052692 GO:0018105 GO:0009737 GO:0009414 GO:0004674 GO:0005986 GO:0010325 GO:0034484 GO:0080167 GO:0005886 GO:0009409 GO:0035556 GO:0009507 GO:0006979 GO:0005524 GO:0019593
TraesCS3A02G092800	GO:0047274 GO:0018107 GO:0052692 GO:0018105 GO:0009737 GO:0009414 GO:0004674 GO:0005986 GO:0010325 GO:0034484 GO:0080167 GO:0005886 GO:0009409 GO:0035556 GO:0009507 GO:0006979 GO:0005524 GO:0019593
TraesCS5D02G300200	GO:0003824 GO:0008152
TraesCS5D02G177700	GO:0005737 GO:0007020 GO:0052692 GO:0009620 GO:0009911 GO:0009965 GO:0016139 GO:0048046 GO:0071555 GO:0046477 GO:0000023 GO:0009505 GO:0043085 GO:0019252
TraesCS3B02G108000	GO:0047274 GO:0018107 GO:0052692 GO:0018105 GO:0009737 GO:0009414 GO:0004674 GO:0005986 GO:0010325 GO:0034484 GO:0080167 GO:0005886 GO:0009409 GO:0035556 GO:0009507 GO:0006979 GO:0005524 GO:0019593
TraesCS2D02G338200	GO:0047274 GO:0052692 GO:0034484 GO:0080167 GO:0009409 GO:0006979
TraesCS7D02G133500	GO:0003824 GO:0008152
TraesCS1D02G444000	GO:0005737 GO:0047274 GO:0005975 GO:0047268 GO:0080167 GO:0004553 GO:0006979
TraesCS3D02G116400	GO:0047274 GO:0005737 GO:0052692 GO:0034484 GO:0080167 GO:0009409 GO:0006979 GO:0009506
TraesCS5A02G173200	GO:0005737 GO:0007020 GO:0052692 GO:0009620 GO:0009911 GO:0009965 GO:0016139 GO:0048046 GO:0071555 GO:0046477 GO:0000023 GO:0009505 GO:0043085 GO:0019252
TraesCS7B02G134600	GO:0047274 GO:0018107 GO:0052692 GO:0018105 GO:0009737 GO:0009414 GO:0004674 GO:0005986 GO:0010325 GO:0034484 GO:0080167 GO:0005886 GO:0009409 GO:0035556 GO:0009507 GO:0006979 GO:0005524 GO:0019593
TraesCS5B02G170600	GO:0005737 GO:0007020 GO:0052692 GO:0009620 GO:0009911 GO:0009965 GO:0016139 GO:0048046 GO:0071555 GO:0046477 GO:0000023 GO:0009505 GO:0043085 GO:0019252
TraesCS3A02G113900	GO:0047274 GO:0005737 GO:0052692 GO:0034484 GO:0080167 GO:0009409 GO:0006979 GO:0009506
TraesCS7B02G035200	GO:0003824 GO:0008152
TraesCS1D02G294900	GO:0034484 GO:0052692 GO:0080167 GO:0016757 GO:0006979
TraesCS4A02G174200	GO:0034484 GO:0052692 GO:0080167 GO:0016757 GO:0006980
TraesCS5D02G271300	GO:0034484 GO:0052692 GO:0080167 GO:0016757 GO:0006981
TraesCS7A02G503400	GO:0034484 GO:0052692 GO:0080167 GO:0016757 GO:0006982

Table S6 Cis-acting elements of *TaRS15-3B* genes.

Cis-acting element name	Number of copies	Function	Classification
CAAT-box	26	Elements common to promoter and enhancer regions	CAAT-box and TATA-box
TATA-box	23	Core promoter elements	CAAT-box and TATA-box
Box 4	1	Elements involved in light reactions	Light responsiveness
C-box	1	Photoreaction-related elements	Light responsiveness
GATA-motif	1	Photoreaction-related elements	Light responsiveness
G-box	7	Photoreaction-related elements	Light responsiveness
GT1-motif	3	Photoreaction-related elements	Light responsiveness
I-box	1	Photo-responsive components	Light responsiveness
ABRE	17	Abscisic acid response element	Phytohomone responsiveness
CGTCA-motif	3	Methyl jasmonate response element	Phytohomone responsiveness
O2-site	1	Methyl jasmonate response element	Phytohomone responsiveness
P-box	1	Erythromycin response element	Phytohomone responsiveness
TGACG-motif	3	Methyl jasmonate response element	Phytohomone responsiveness
TGA-element	1	Growth factor response element	Phytohomone responsiveness
as-1	3	Root-specific expression element	Plant growth and development
motif I	1	Root-specific expression elements	Plant growth and development
MSA-like	1	Cis-acting elements involved in cell cycle regulation	Plant growth and development
ARE	1	Cis-regulatory elements essential for anaerobic induction	Stress responsiveness
DRE core	4	Drought, hypersalinity and low temperature responsive cis-acting elements	Stress responsiveness
GC-motif	1	Enhancer-like elements involved in hypoxia-specific induction	Stress responsiveness
MBS	1	MYB-binding site, involved in drought induction	Stress responsiveness
MBSI	2	MYB-binding site, involved in drought induction	Stress responsiveness
MYB	7	Drought abiotic stress	Stress responsiveness
MYB	1	Drought abiotic stresses	Stress responsiveness
MYC	4	Abiotic stresses such as drought and salt	Stress responsiveness
STRE	2	Stress response elements	Stress responsiveness

Table S7 The primers used in this study

Target segments	Forward sequence（5'-3'）	Reverse sequence（5'-3'）
TaRS3-1A	ATCCTGATATGCTCGAAGTTGG	TTATGGTCTTTGTCTGCGGG
TaRS10-2A	GAGAGGCTGGTTGATCTGAAG	CCTCCCCAATACCCAAGTAAAG
TaRS13-3A	ATCGGCAAGCTGCTCGACACGC	GAGGAGGAGCACGTAGGGACG
TaRS15-3B	CTGGTTGACACGAGGTTCAT	CATCATCTGGGTCTCGTTCTC
TaRS17-3D	ACTTCCAGGGGCTGACGGAG	AGAGGGAGTTGTAAGCGCAG
TaRS18-3D	GAGAGGCTGGTTGATCTGAAG	CCTCCCCAATACCCAAGTAAAG
TaCAT3-F	CGCTCACTGATGCTCGTGTTACC	TCCATCTACATGCTCGGCTTAAT CTTG
TaSODI-F	TCCTTTGACTGGCCCTAATG	CTTCCACCAGCATTTCCAGT
TaPODA1	AGCACACAAGGAGAGAGGAG	AAGAGGCACGCGGTATCG
TaLEA7-F	GTCGAGAGCAAGGACCAGAC	CCTGTCCTGCGTGTACTGC
TaP5CS-F	GACAAGTCCCGTGTTGGTAGAG	CGTGCAGCAACAGCCATTT
TaDREB1-F	GAACCACTCCCTCCATCTC	TTCACAGCACCGACCAA
β-actin	AAGAGTCGGTGAAGGGGACT	GGAAAAGCTGAACCGAGACTG
TaRS15-3B Silent fragment linker	TAGCTAGCTGATTAATTAAGATC GTGGACAAGTTCGGGT	TTGCTAGCTGAGCGGCCGCTCCT GGAACTTGATGAGCCG
TaRS15-3B Silent fragment	GATCGTGGACAAGTTCGGGT	TCCTGGAACTTGATGAGCCG

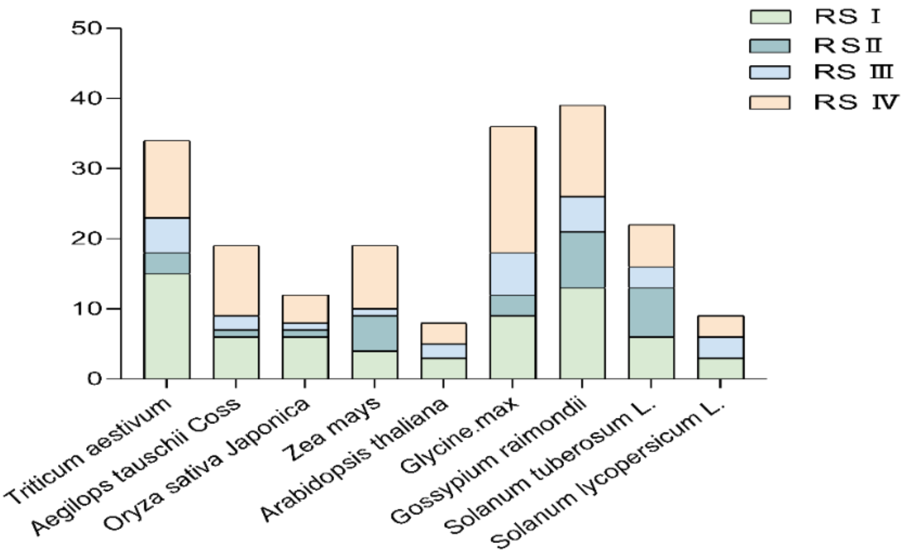

Figure S1 The number of *RS* genes found in each subfamily of Triticum aestivum、Aegilops tauschii Coss、Oryza sativa Japonica、Zea mays、Arabidopsis thaliana、Glycine.max、Gossypium raimondii 、Solanum tuberosum L. and Solanum lycopersicum L.

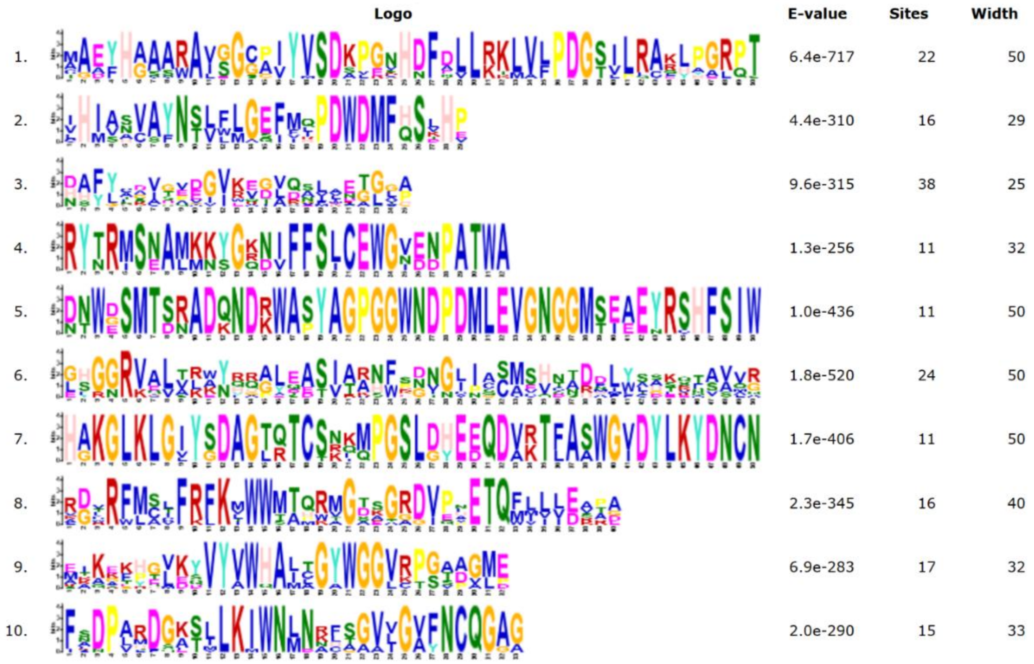

Figure S2 Information of the identified motifs in TaRS proteins.

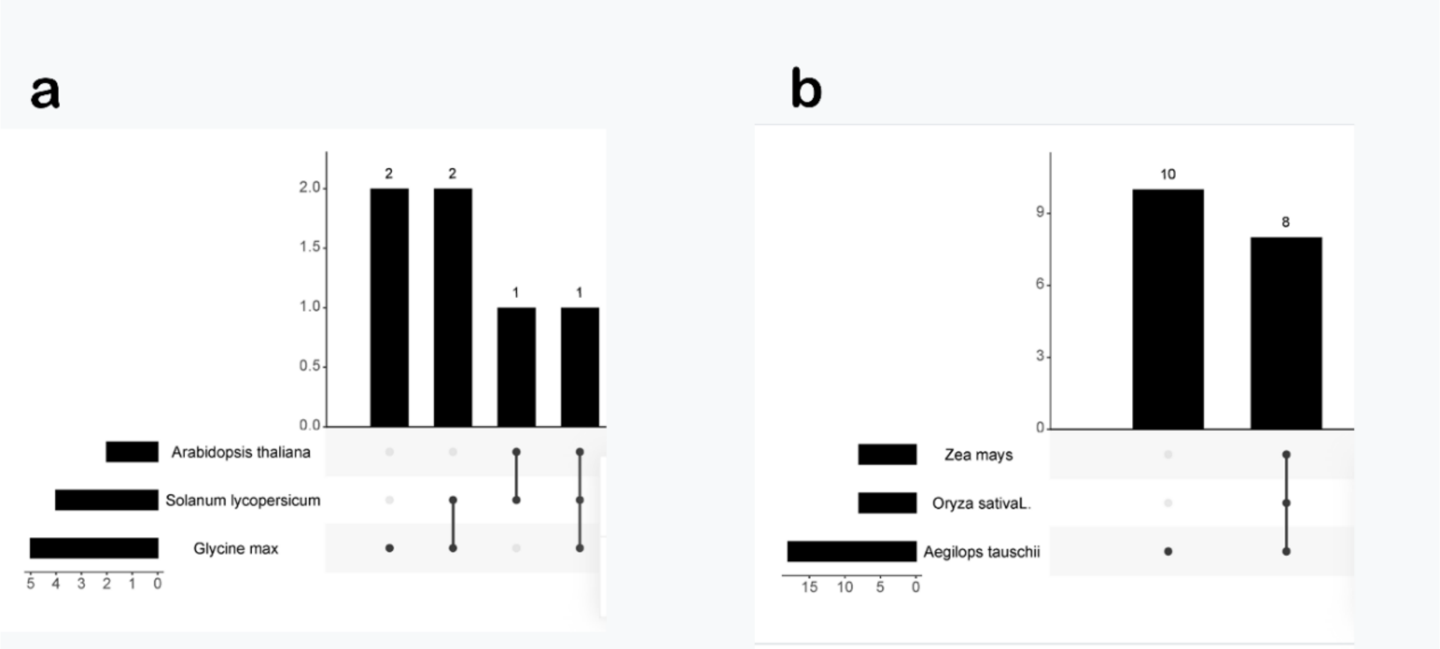

Figure S3 UpSet diagram of non redundant genes between wheat, monocotyledonous plants, and dicotyledonous plants

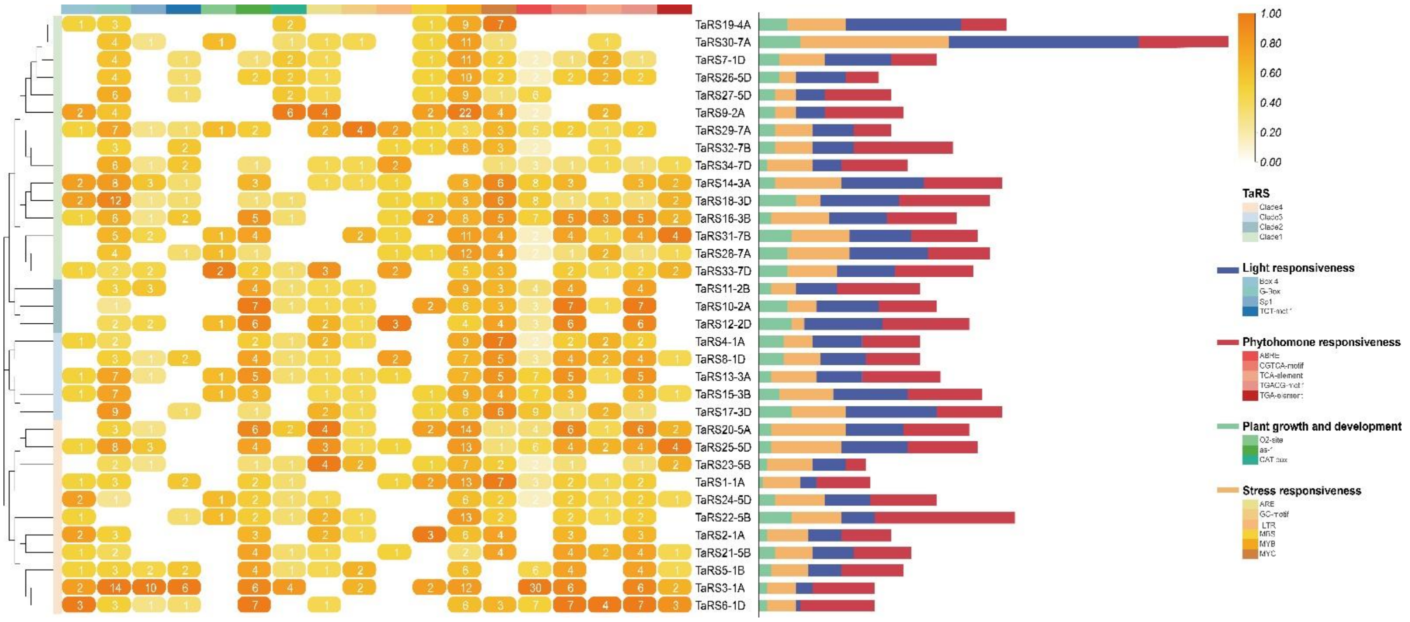

Figure S4 Cis-acting elements of wheat *TaRS* genes. a) Heatmap of cis-acting elements per *TaRS* genes numbers; deep colors indicate the more cis-acting elements; white means no cis-acting elements. The column icon on the right indicates the number of cis-acting elements for each *TaRS* gene. Different types of cis-acting elements are shown in different colors.

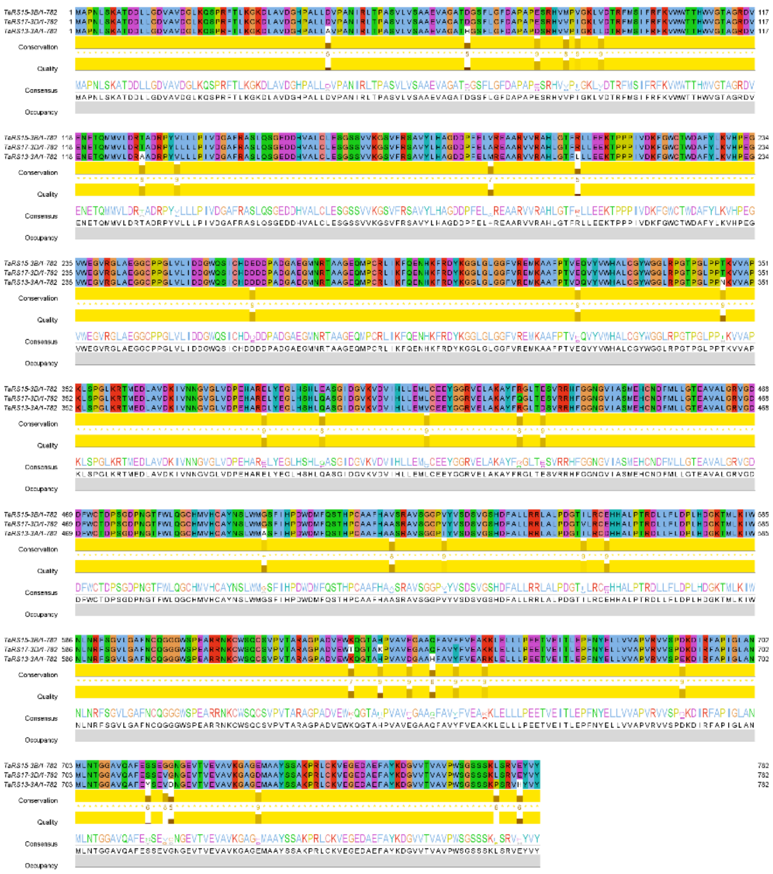

Figure S5 TaRS15-3B Protein Multiple Sequence Alignment

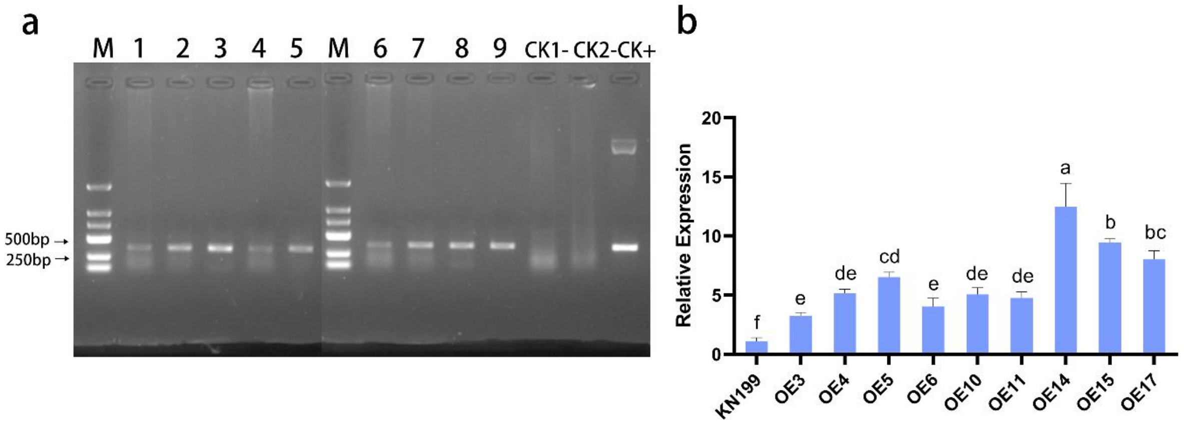

Figure S6 Generation and identification of overexpressing TaRS15-3B wheat: (a) PCR assay of overexpressing TaRS15-3B wheat， M: DL2000; 1-9: for OE3, 4, 5, 6, 10, 11, 14, 15, 17 respectively; CK1-:KN199; CK2-:H2O; CK+:positive plasmid t; (b) qPCR assay of overexpressing TaRS15-3B wheat

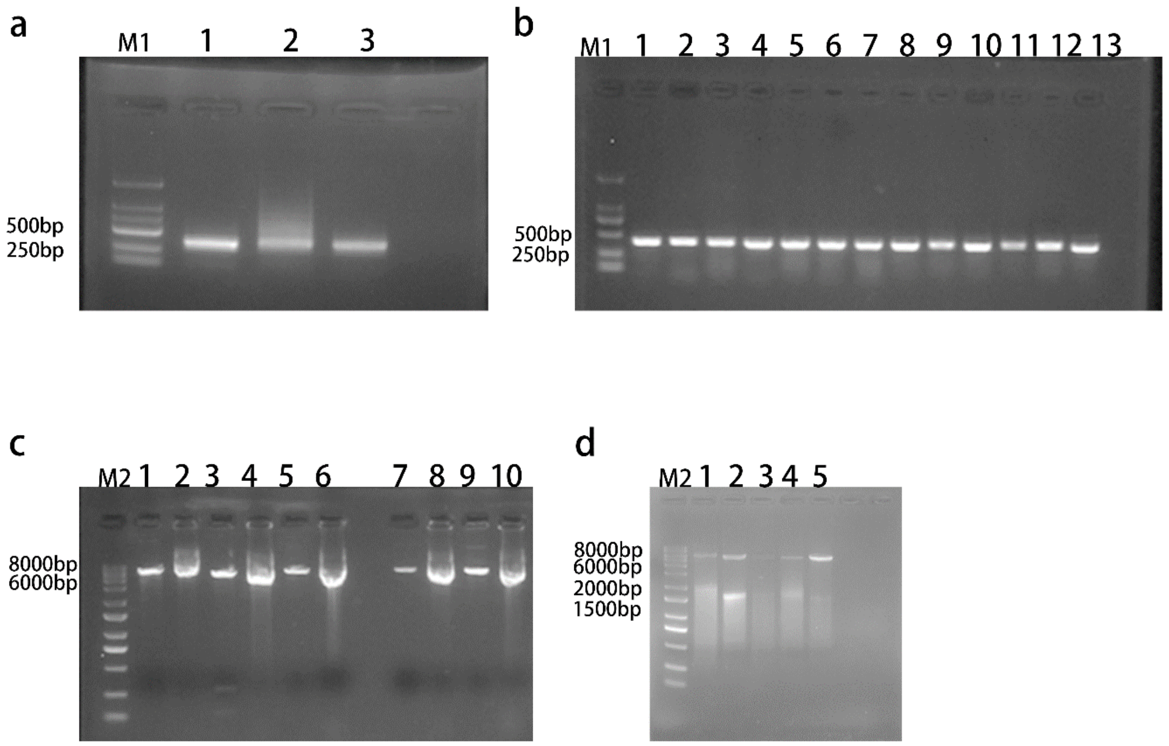

Figure S7 Construction of BSMV-VIGS vector: (a) acquisition of silenced fragments; (b) Construction of BSMV: γ-*TaRS15-3B* vector: (c) vector linearization; (d) : in vitro transcription
